# Determinants of Length of Stay for Medical Inpatients Using Survival Analysis

**DOI:** 10.3390/ijerph21111424

**Published:** 2024-10-26

**Authors:** Jaekyeong Kim, Haegak Chang, Seiyoung Ryu, Ilyoung Choi, Angela Eunyoung Kwon, Haeyong Ji

**Affiliations:** 1 School of Management, Kyung Hee University, Seoul 02447, Republic of Korea; jaek@khu.ac.kr (J.K.); hkc@khu.ac.kr (H.C.); 2 Department of Bigdata Analytics, Kyung Hee University, Seoul 02447, Republic of Korea; rsy22@khu.ac.kr; 3 Division of Business Administration, Seo Kyeong Uiversity, Seoul 02713, Republic of Korea; iychoi@skuniv.ac.kr; 4 Sauder School of Business, University of British Columbia, Vancouver, BC 2053, Canada; angela.kwon@sauder.ubc.ca; 5 Department of Management, Graduate School, Kyung Hee University, Seoul 02447, Republic of Korea

**Keywords:** survival analysis, Kaplan–Meier survival analysis, Cox proportional hazards model, health checkup cohort DB, length of stay, medical data

## Abstract

With the increase in insured patients and an aging population, managing the length of stay (LOS) for inpatients has become crucial for controlling medical costs. Analyzing the factors influencing LOS is necessary for effective management. Previous studies often used multiple or logistic regression analyses, which have limitations such as unmet assumptions and the inability to handle time-dependent variables. To address these issues, this study applied survival analysis to examine the factors affecting LOS using the National Health Insurance Service (NHIS) sample cohort data from 2016 to 2019, covering over 4 million records. We used Kaplan–Meier survival estimation to assess LOS probabilities based on sociodemographic, patient, health checkup, and institutional characteristics. Additionally, the Cox proportional hazards model controlled for confounding factors, providing more robust validation. Key findings include the influence of age, gender, type of insurance, and hospital type on LOS. For instance, older patients and medical aid recipients had longer LOS, while general hospitals showed shorter stays. This study is the first in Korea to use survival analysis with a large cohort database to identify LOS determinants. The results provide valuable insights for shaping healthcare policies aimed at optimizing inpatient care and managing hospital resources more efficiently.

## 1. Introduction

With the surge in insured patients and an aging population, the sustainability of expanding medical coverage has become a pressing concern. Vulnerable groups, including medically insured patients, the homeless, and refugees, often experience extended hospital stays due to the lack of family support, limited access to medical services, and financial constraints. These factors impact the length of stay (LOS) in hospitals, making its rational management beneficial for both patients and healthcare institutions.

From an institutional perspective, reducing the LOS can improve the turnover rate of hospital beds, allowing for a greater influx of new patients and increasing revenue. From a patient perspective, timely discharge following acute treatment promotes recovery through outpatient care, helping to reduce medical expenses. Therefore, effectively managing the LOS through a careful analysis of the factors influencing it is essential for optimizing healthcare delivery.

Previous studies have applied various methodologies, such as multiple regression, logistic regression, and machine learning, to identify determinants affecting the LOS [1,2,3,4], and to predict LOS [5,6,7,8,9,10,11]. However, these approaches often fail to account for censored data or the survival period of inpatients. For instance, multiple regression models do not consider the censored nature of the data despite time being a dependent variable, and logistic regression models overlook the survival period, despite survival being directly linked to the outcome. To address these limitations, it is crucial to consider both the censored data and the survival period of inpatients when analyzing the determinants of LOS.

The aim of this study is to identify key factors influencing the LOS for inpatients, taking into account the censored nature of the data and the survival period. We employ survival analysis techniques to achieve this goal. Specifically, we use Kaplan–Meier survival estimation to analyze the trend of rehospitalization probability over time, and the Cox proportional hazards model to compare factors affecting the probability of discharge.

Referring to variables used in existing studies, we selected factors from the cohort database provided by the National Health Insurance Service (NHIS) of Korea. Data analysis was performed using the R statistical program (version 3.7.6) in the NHIS virtual environment. Based on the findings, healthcare institutions can improve resource management and reduce unnecessary expenses by optimizing LOS management.

## 2. Research Background

### 2.1. Length of Stay (LOS) Analysis

Length of stay (LOS), the period during which inpatients are hospitalized, is a key indicator for the efficient management of medical institutions and the reduction in patients’ medical expenses. In Korea, the average LOS decreased from 19.1 days in 2018 to 17.9 days in 2019, but increased back to 19.1 days in 2020. Notably, as of 2020, the average LOS in Korea is 10.8 days longer than the OECD average of 8.3 days.

Previous studies have investigated the determinants of LOS, as summarized in Table 1. Most of these studies utilized multiple regression analysis, considering socio-demographic, disease, and institutional characteristics as independent variables to examine LOS.

As such, most prior studies analyzing the determinants of LOS have focused on subjects from specific groups or disease categories. However, although data regarding LOS are considered count data, these studies have limitations as they do not account for this aspect. Therefore, this study aims to scrutinize the determinants affecting inpatients’ LOS using survival analysis for count data.

### 2.2. Survival Analysis

Survival analysis is a method widely used in biology and medicine, which utilizes censored data containing information such as patients’ survival and death and assesses differences in the elapsed time to an event of interest [14,15,16]. Here, censored data refer to data where the occurrence of an event is unknown from the beginning of the study to its end. Since survival analysis is a statistical approach that estimates the time between two events of interest, it differs explicitly from other approaches such as regression and logistic regression, as demonstrated in Table 2. While regression considers time as a dependent variable, it is limited by its inability to account for censored data. On the other hand, logistic regression can only include an event, such as whether one has died or been hospitalized, as a dependent variable, but it cannot consider time in its analysis.

In fact, survival analysis can be conducted using three types of methods: non-parametric, semi-parametric, and parametric methods. First, a non-parametric method does not require an assumption that the data follow a specific probability distribution. Second, a semi-parametric method also does not require an assumption regarding data distribution but estimates regression coefficients. Lastly, a parametric method assumes that the data follow a particular distribution, such as the Weibull distribution, with respect to survival time t.

In this study, we decided to use the Kaplan–Meier estimation, a non-parametric method, along with the log rank test, as our health checkup cohort DB does not conform to any specific type of distribution, including the Weibull distribution.

The Kaplan–Meier estimation assumes that events occur independently of one another and calculates survival probabilities from one interval to the next, under the assumption that censoring is independent of the survival time [17]. These probabilities can be illustrated in a survival plot [17].

The log rank test compares time-to-event distributions across two or more independent groups using a Chi-square test of the time occurrence between observed and expected counts. This test is particularly used to validate the null hypothesis that no significant difference exists in the survival curves between the groups being compared.

Additionally, our study incorporates the Cox proportional hazards model, a multivariate regression method that tests the significance of various time-relevant predictors, assuming a log-linear relationship between the survival function and the variables [18].

## 3. Methodology

### 3.1. Comprehensive Framework

**Figure 1:**
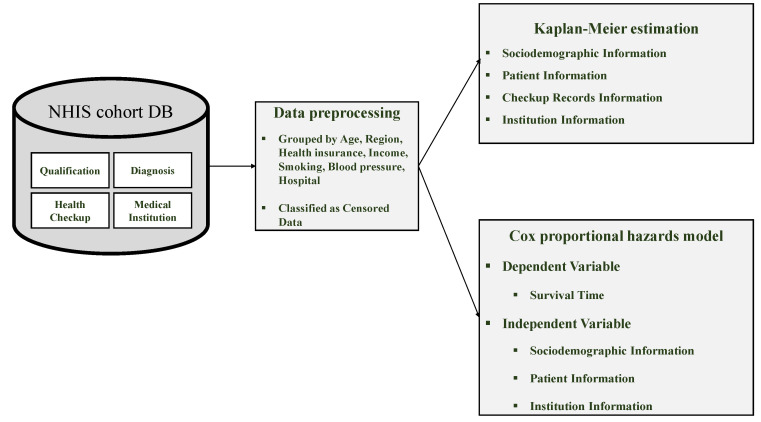
Comprehensive framework for analyzing determinants of LOS.

The purpose of this study was to examine the determinants affecting the length of stay (LOS) of inpatients. We present the comprehensive framework of our research in Figure 1, which can be categorized into three main phases: (1) data collection, (2) data preprocessing, and (3) survival analysis.

During the data collection phase, we gathered the health checkup cohort DB. In the data preprocessing phase, we grouped and classified the censored data. Finally, for the survival analysis phase, we investigated the main determinants of LOS using both Kaplan–Meier estimation and the Cox proportional hazards model.

### 3.2. Data

To identify the variables affecting inpatients’ LOS, this study used the four-year health checkup cohort DB from 2016 to 2019, provided by the Korean National Health Insurance Services. This health checkup cohort DB is a sample study database composed of medical records for examinees aged 40 to 79, which are used for various medical analyses. The data are categorized into four tables, which are explained in Table 3.

### 3.3. Data Preprecessing

Based on previous studies investigating LOS determinants, we classified the variables from the health checkup cohort DB into four categories, as shown in Table 3. Of the 22 variables used for preprocessing, 15 were grouped into sociodemographic, patient, health checkup, and institutional categories for LOS analysis.

First, we used the ‘Qualification and Income Range’ table to select variables related to sociodemographic characteristics, such as gender, age, and residing area, and patient features like insurance type, income quantile, and severity of disability. The date of death was treated as censored data. Second, from the ‘Diagnosis’ table, we extracted inpatient records and used LOS as the dependent variable, focusing only on main diagnoses. Third, from the ‘Health Checkup’ table, we selected variables like smoking status, BMI, systolic, and diastolic blood pressure. Lastly, from the ‘Medical Institution’ table, we used variables related to the institution type, number of beds, and whether CT, MRI, or PET scanners were available.

As shown in Table 4, data preprocessing for survival analysis involved handling missing values, merging tables, extracting feature values, and identifying censored data. The key steps are as follows.

First, missing values were addressed using features like health insurance type, disability severity, diagnostic results, and date of death. Missing insurance data were excluded, and missing disability values were marked as ‘no disability’. Missing death dates were treated as censored data, indicating survival or unknown status.

Second, feature preprocessing involved grouping variables before analysis. Age was converted to numeric values and grouped in 10-year increments. Subjects under 50 were excluded, and regions were grouped into Seoul, Gyeonggi-do, and others. Insurance types were reduced to three categories: medical, region, and workplace insurees. Income quantiles were grouped into three (1–3, 4–6, 8–10). Smoking status was simplified into two groups, smoker and non-smoker, while BMI was categorized into four groups, underweight, normal, overweight, and obese, based on KDCA standards. Blood pressure was also grouped into hypotension, normal, and hypertension. The ‘Medical Institution’ table grouped institutions by type, including general hospitals, clinics, and care hospitals, and classified bed numbers.

Third, feature extraction was limited to inpatient records and only main diagnoses were used. Finally, the ‘date of death’ feature classified those alive at discharge as censored data.

In total, 15 variables were used to analyze LOS determinants across 228,670 preprocessed cases, consisting of 227,644 discharge records and 1026 censored cases. The details of data preprocessing are presented in Table 5.

## 4. Results

This study used survival analysis to investigate the determinants of inpatients’ length of stay (LOS). A total of 3,228,933 records were analyzed to examine how twelve different features affect the death rate.

### 4.1. General Characteristics of Study Subjects

The general characteristics of the study subjects are presented in Table 6.

### 4.2. Kaplan–Meier Estimation

**Figure 2:**
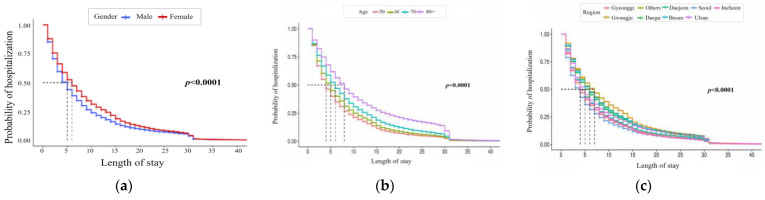
Kaplan–Meier curve of LOS by sociodemographic feature. The three charts represent (**a**) gender, (**b**) age, and (**c**) region (from left to right).

This study uses Kaplan–Meier estimation to analyze how sociodemographic, patient, health checkup, and institutional features affect LOS over time. First, Figure 2 shows estimates for sociodemographic features, including gender, age, and city/province. Females have a higher probability of being hospitalized than males, indicating a lower discharge probability. Hospitalization probability also varies by age and region, with Seoul having the lowest and Gwangju the highest rates.

**Figure 3:**
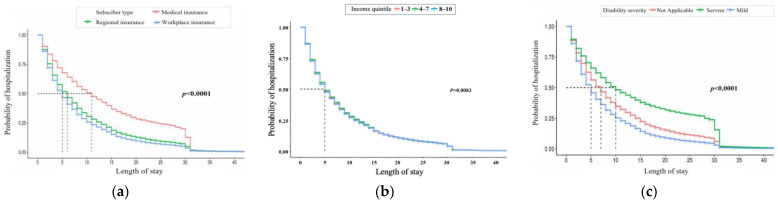
Kaplan–Meier curve of LOS by patient feature. The three charts represent (**a**) insurance status, (**b**) income quantile, and (**c**) disability (from left to right).

Figure 3 presents Kaplan–Meier curves for patient features like health insurance type, income quantile, and disability severity. Medical insurees show a higher probability of admission than regional and workplace insurees. Admission probabilities also differ across income quantiles, and individuals with severe disabilities have a higher likelihood of hospitalization compared to those with mild symptoms.

**Figure 4:**
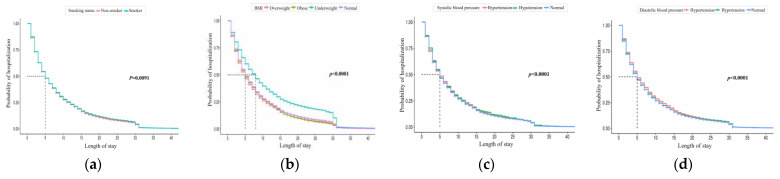
Kaplan–Meier curve of LOS by checkup record feature. The four charts represent (**a**) smoking status, (**b**) BMI, (**c**) systolic blood pressure, and (**d**) diastolic blood pressure (from left to right).

Figure 4 shows the results for health checkup features, where non-smokers and smokers differ in hospitalization probabilities. Underweight patients have the highest hospitalization rates for BMI, and hospitalization probabilities vary by systolic and diastolic blood pressure levels.

**Figure 5:**
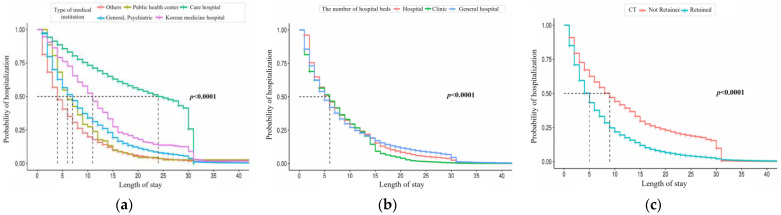

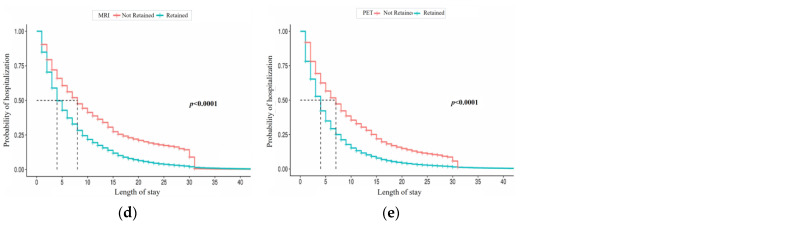
Kaplan–Meier curve of LOS by institution feature. The five charts represent (**a**) type of medical institution, (**b**) number of hospital beds, (**c**) CT, (**d**) MRI, and (**e**) PET (from left to right).

Finally, Figure 5 shows estimates obtained by institutional features. Care hospitals have the highest hospitalization probability, and differences are observed in the number of hospital beds. Institutions lacking CT, MRI, or PET scanners show a higher probability of hospitalization.

### 4.3. Cox Proportional Hazards Model

**Figure 6:**
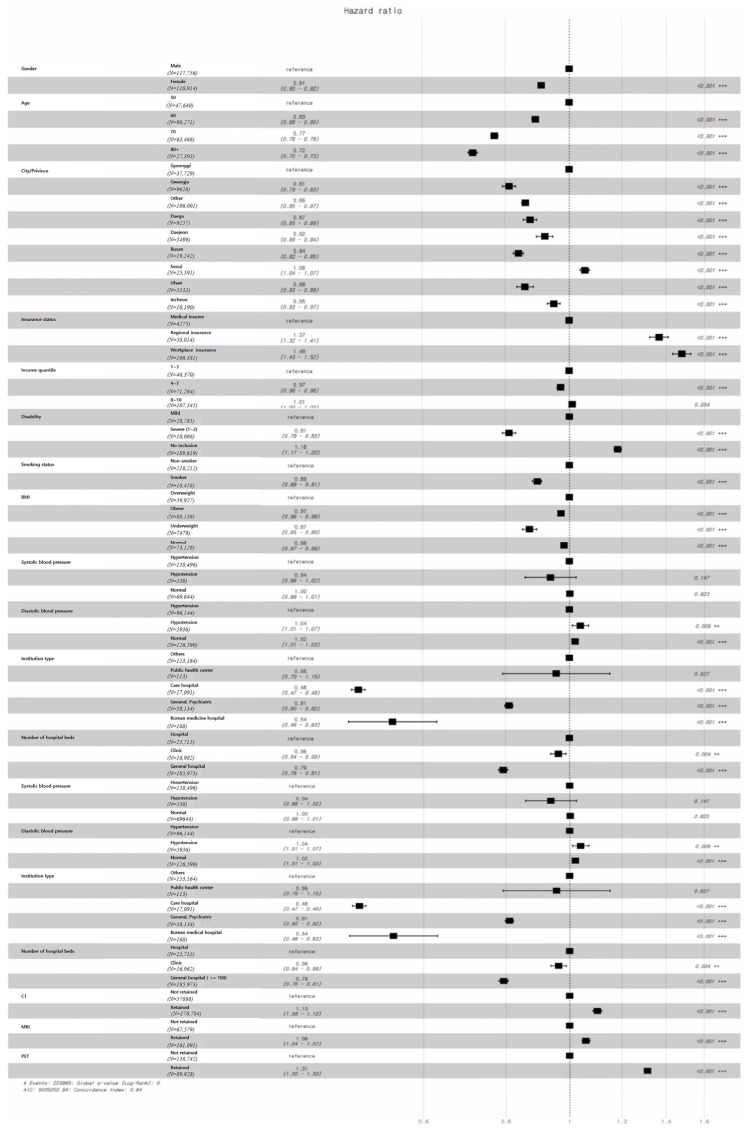
Results of the Cox proportional hazards model for LOS.

The variables affecting the probability of discharge were identified using the Cox proportional hazards model, as summarized in Figure 6.

In terms of gender, the probability of discharge for males is 0.91 times lower than for females. For age, the probability of discharge decreases for those in their 50s to 80s. Compared to those in their 50s, the discharge probability is 0.89, 0.77, and 0.72 times for inpatients in their 60s, 70s, and 80s, respectively, with those aged 80 and above having the highest probability of hospitalization.

Looking at regional features, the discharge probabilities for Gwangju, Daegu, Daejeon, Busan, Seoul, Incheon, and other regions range from 0.81 to 1.06 times that of Gyeonggi, with Gwangju showing the most significant positive impact on LOS, and Seoul having a negative impact.

Regarding insurance type, the probability of discharge for patients with workplace insurance and regional insurance is 1.48 and 1.37 times higher than that for medical insurees, respectively. Patients in the 4–7 income quantiles have a discharge probability 0.97 times that of the 1–3 quantile group, while no significant difference was observed between the 8–10 and 1–3 quantiles. Patients without disabilities have a discharge probability 1.18 times higher than those with mild symptoms, while those with severe symptoms have a lower probability of 0.81 times, indicating a positive impact of severe symptoms on LOS.

For health checkup records, smokers have a discharge probability 0.89 times that of non-smokers. In terms of BMI, underweight, obese, and normal patients have discharge probabilities of 0.87, 0.97, and 0.98 times that of overweight patients, showing the influence of BMI on LOS. Systolic blood pressure showed no significant difference, but for diastolic blood pressure, patients with hypotension and normal levels had discharge probabilities of 1.04 and 1.02 times that of those with hypertension.

Lastly, the probability of discharge for care hospitals, Korean medicine hospitals, and psychiatric hospitals is 0.48, 0.54, and 0.81 times that of other institutions. However, no significant difference was found between public healthcare centers and other institutions. Hospitals with more beds and those possessing medical devices such as CT, MRI, and PET scanners had higher discharge probabilities, indicating their influence on LOS.

The results derived from the Cox proportional hazards model largely align with previous studies. However, as shown in Table 7, discrepancies were observed in the number of hospital beds. Specifically, the probability of discharge in hospitals was found to be higher than that in clinics and general hospitals.

## 5. Discussion

This study explored key determinants influencing inpatients’ length of stay (LOS) using Kaplan–Meier estimation and Cox proportional hazards models. These methods complement each other, with Kaplan–Meier capturing univariate trends and Cox models providing multivariate insights. Some findings align with previous research, while others offer new perspectives.

Sociodemographic Characteristics: Male patients showed higher discharge probabilities than females, consistent with studies suggesting women experience more complex health conditions [10,19]. Older patients demonstrated longer stays, in line with the findings reported by [20]. Patients in Seoul had shorter stays, likely due to stricter discharge policies [10].

Patient Characteristics: Insurance type significantly impacted LOS, with medical insurees showing the longest stays, as reported by [21].

However, contrary to earlier studies [22], middle-income patients (quantiles 4–7) had longer stays. Severe disabilities were linked with longer stays, reinforcing findings that complex cases require prolonged care [23].

Health Checkup Records: Smoking status was a strong predictor of LOS, with smokers experiencing longer stays, supporting findings on the impact of smoking-related illnesses [24]. Underweight patients had the most prolonged stays, possibly due to heightened infection risks [25,26]. Diastolic hypertension, rather than systolic pressure, was associated with longer stays, echoing the trends identified by [27].

Institutional Characteristics: Care hospitals had the most significant effect on LOS, consistent with their role in long-term care [28]. However, contrary to earlier studies [10], we found that general hospitals did not retain patients longer than smaller institutions. Furthermore, the absence of advanced diagnostic equipment, such as CT, MRI, and PET scanners, was associated with longer stays, likely due to delayed diagnosis [29].

Implications for Practice: Our findings highlight the importance of addressing diverse factors affecting LOS to improve hospital resource management. For efficient care delivery, healthcare providers should consider tailored strategies for older adults, smokers, and patients with chronic illnesses or disabilities. Policymakers could also explore interventions to optimize discharge processes and enhance the availability of diagnostic equipment in smaller institutions.

## 6. Conclusions

This study identified key determinants influencing inpatients’ LOS using survival analysis methods. By leveraging Kaplan–Meier and Cox proportional hazards models, this research offers insights into how various sociodemographic, patient, health checkup records, and institutional factors shape LOS. The findings indicate that male gender, younger age, medical insurance type, and institutional features like equipment availability play significant roles in determining LOS. These findings have practical implications for healthcare policy and management. Specifically, they underscore the need for clear guidelines regarding patient admission to care hospitals and the potential benefits of targeted rehabilitation services for vulnerable groups. Reducing LOS can enhance hospital revenue while lowering patient costs, thereby contributing to the sustainability of health insurance systems.

The study also suggests future research directions, such as deeper exploration of regional differences in LOS and expanding analyses to include other patient demographics. Further investigations should continue to refine our understanding of LOS determinants, incorporating newer methods and data sources to inform hospital policies and improve patient care outcomes.

## Figures and Tables

**Table 1:** Previous literature on determinants of LOS.

Researchers	Research Subjects	Methodology	Feature
[5]	Long-term insured inpatients	Multiple linear regression	Sociodemographic features (gender, age, types of medical insurance) Disease features (ratio of chronic illness, patient categorization) Admission features (Type 1: hospitalized for one year in a care hospital only; Type 2: hospitalized for a long duration in various institutions including a care hospital; Type 3: Hospitalized for a long duration in institutions other than a care hospital)
[7]	Hepatic cirrhosis patients	Poisson regression	Gender, age, type of medical insurance, diagnostic result
[8]	Total knee replacement or hip arthroplasty patients	Multivariate linear regression	Gender, age, year of surgery, diagnoses recorded in CPRD, RCS Charlson score, IMD quintiles
[9]	Neonates with cardiac surgery	Path analysis	Age at surgery, postoperative feeding, sepsis, birth distance from the surgical hospital, preoperative feeding, major genetic anomaly, necrotizing enterocolitis, specialist
[10]	Total knee arthroplasty patients	Multiple linear regression	Sociodemographic features (gender, age, type of medical insurance) Disease feature (severity, 17 groups of diagnosis severity) Institution features (location, the number of hospital beds)
[11]	Patients with prolonged LOS at emergency department in a tertiary care center	Multinomial/binomial logistic regression	Gender, age, hospitalization route (arrival by ambulance), severity classification, expertise in treatment, diagnostic test, consultation, number of comorbidities, triage types and numbers, number of patients in emergency department (ED) at the time of ED registration
[12]	Total knee arthroplasty patients	Multiple linear regression Multiple logistic regression	Sociodemographic features (gender, age, types of medical insurance) Disease features (severity, matter of depression) Medical institution features (location, the number of hospital beds)
[13]	Early-/late-stage cancer elderly patients	Multiple linear regression	Gender, method of payment, admission route, sub diagnosis, matter of undergoing a surgical procedure for main diagnosis, institution location, the number of hospital beds

**Table 2:** Comparison of methodologies used in survival analysis.

Category	Characteristics	Limitation
Regression	Dependent variable: Time	Cannot consider the presence of censored data
Logistic regression	Dependent variable: Event	Cannot consider time
Survival analysis	Can consider both time and the presence of censored data	

**Table 3:** Health checkup cohort DB.

Table	Description
Qualification	Includes socio-demographic information (gender, age, residential area, income range, insurance type) of a health checkup examinee or information about the matter of death or impairment
Diagnosis	Includes medical records (main diagnosis information, prescription history, cost-related information, admission records, the department, etc.) of a health checkup examinee
Health Checkup	Includes checkup records (lab value, past medical history, hereditary conditions, lifestyle, etc., retrieved from survey questionnaires) of a health checkup examinee
Medical Institution	Includes information of a medical institution (location, the number of doctors and hospital beds, medical infrastructure) attended by a health checkup examinee

**Table 4:** Features in health checkup cohort DB *.

Table	Feature Code	Feature Description	Purpose of Use
Common	STD_YYYY	Year between 2016 and 2019	Merging between tables
	PERSON_ID	Alternative identification ID for resident registration number	
	YKIHO_ID	Alternative identification ID for institution registration number	
Qualification	SEX	1: Male, 2: Female	Sociodemographic feature
	AGE	Patient’s age in a corresponding year	
	SIDO	City/Province code	
	IPSN_TYPE_CD	Type of insurance	Patient feature
	CTRB	Income quantile (1~10)	
	DFAB_GRD_CD	No inclusion, severe, mild level of disability	
	DTH_MDY	Date of death	Censoring
Diagnosis	FORM_CD	Type of treatment (inpatient, outpatient)	Preprocessing
	VSCN	Days of receiving treatments	Dependent variable
	SICK_DIV_TYPE_CD	Diagnosis classification (main diagnosis, sub diagnosis)	Preprocessing
Health Checkup	SMK_STAT_TYPE_RSPS_CD	Smoking status	Health checkup records feature
	BMI	Body Mass Index	
	BP_HIGH	Systolic blood pressure	
	BP_LWST	Diastolic blood pressure	
Medical Institution	INST_CLSFC_CD	Type of medical institutions	Institution feature
	SICKBED_CNT	The number of hospital beds	
	CT_CNT	Retention of CT	
	MRI_CNT	Retention of MRI	
	PET_CNT	Retention of PET	

* Feature code is directly retrieved from the data provided by NHIS.

**Table 5:** Preprocessed results by feature (LOS determinant analysis).

Category	Feature	Preprocessed Results
Sociodemographic Information	Gender	[Grouping] Male/Female
	Age	[Feature Preprocessing] Exclude <50 [Grouping] 10 year unit
	City/ Province	[Grouping] Seoul/Gyeonggi/Daegu/Daejeon/Busan/Ulsan/ Gwangju/Incheon/Others
Patient Information	Insurance status	[Grouping] Workplace/Region/Medical Insurees
	Income quantile	[Grouping] 1~3/4~7/8~10
	Disability	[Grouping] No inclusion, Severe, Mild
Checkup Record Information	Smoking status	[Grouping] Non-smoker/Smoker
	BMI	[Grouping] Underweight (<18.5), Normal (<23), Overweight (<25), Obese (≥25)
	Systolic Blood Pressure	[Grouping] Hypotension (<90), Normal (<120), Hypertension (≥120)
	Diastolic Blood Pressure	[Grouping] Hypotension (<60), Normal (<80), Hypertension (≥80)
Institution Information	Institution type	[Grouping] Public Health Center/Care Hospital/General, Psychiatric Hospital/Korean Medicine Hospital/Others
	Number of Hospital Beds	[Grouping] Clinic (≤30), Hospital (<100), General Hospital (≥100)
	CT	[Grouping] Included/Not included
	MRI	
	PET	

**Table 6:** General characteristics of hospitalized inpatients.

Category	Feature		Number of Patients	Number of Censored Data	Number of Dead
			N	N	N
Sociodemographic Information	Gender	Male	117,756	712	117,044
		Female	110,914	314	110,600
	Age	50~	47,640	61	47,579
		60~	90,271	184	90,087
		70~	63,466	391	63,075
		80~	27,293	390	26,903
	City/Province	Seoul	25,591	108	25,483
		Gyeonggi	37,729	153	37,576
		Daegu	9257	58	9199
		Daejeon	5499	26	5473
		Busan	19,242	68	19,174
		Ulsan	5533	14	5519
		Gwangju	9628	27	9601
		Incheon	10,190	26	10,164
		Others	106,001	546	105,455
Patient Information	Insurance status	Medical insuree	4275	14	4261
		Regional insurance	58,014	314	57,700
		Workplace insurance	166,381	698	165,683
	Income quantile	1~3	216	46,154	46,370
		4~7	285	70,979	71,264
		8~10	513	106,832	107,345
	Disability	No inclusion (Normal)	787	189,032	189,819
		Mild	64	10,002	10,066
		Severe	175	28,610	28,785
Checkup Record Information	Smoking status	Non-smoker	925	209,287	210,212
		Smoker	101	18,357	18,458
	Body Mass Index (BMI)	Underweight	122	7357	7479
		Normal	434	72,694	73,128
		Overweight	234	59,693	59,927
		Obese	236	87,900	88,136
	Systolic blood pressure	Hypotension	6	524	530
		Normal	293	69,351	69,644
		Hypertension	727	157,769	158,496
	Diastolic blood pressure	Hypotension	29	5907	5936
		Normal	562	126,028	126,590
		Hypertension	435	95,709	96,144
Institution Information	Type of medical institution	Public health center	1	112	113
		Care hospital	221	16,870	17,091
		General, psychiatric	73	58,061	58,134
		Korean medicine hospital	0	168	168
		Others	731	152,433	153,164
	The number of hospital beds	Clinic	8	16,974	16,982
		Hospital	39	25,676	25,715
		General hospital	979	184,994	185,973
	CT	Not included	278	57,608	57,886
		Included	748	170,036	170,784
	MRI	Not included	328	160,393	160,721
		Included	698	67,251	67,949
	PET	Not included	511	138,231	138,742
		Included	515	89,413	89,928
Total			228,670	1026	227,644

**Table 7:** Hazard ratio by number of hospital beds.

Feature		Hazard Ratio	*p*-Value
Number of hospital beds	Hospital (reference)		
	Clinic	0.96	0.004
	General hospital	0.79	<0.001

## Data Availability

Data sharing is not applicable to this article.
